# Supplementary material for: Patterns of Intron Gain and Loss in Fungi
Source: PLoS Biol. 2004 Nov 30;2(12):e422. doi: 10.1371/journal.pbio.0020422 (PMC532390; doi:10.1371/journal.pbio.0020422)
Supplement: Table S1 — Also available at http://genes.mit.edu/NielsenEtAl/. (4.3 MB ZIP). [file pbio.0020422.st001.zip › NielsenEtAl/html/302.html]

AN2528.1.NCU09976.1.MG07677.1.FG04848.1


```
 CLUSTAL W (1.82) Multiple Sequence Alignments - Introns Inserted


Sequence 1: MG07677.1	259 aa
Sequence 2: FG04848.1	257 aa
Sequence 3: NCU09976.1	269 aa
Sequence 4: AN2528.1	245 aa
Alignment Length: 274 aa
Number Identitical Residues: 88 aa
Alignment Score (without introns) 4536


MG07677.1 	MQLPTSFFAGALGALLLPQQVL--------ATIYLCG~DSTMARSGANDGQTDG~WGNHF
NCU09976.1	MRFSSTALIASLAAASVDARSVNPCDKRATPTVYLAG~DSTTAKTG---GLLMG1WGEYL
FG04848.1 	MKFTVSFAALAAGAFQAVAG----------QTVYFAG1DSTMARSGANDGATDG~WGNYI
AN2528.1  	---MKSIALTSLSLLPSALA----------QTIYLAG~DSTMASST------PG1WGDYI
          	     :    : .                  *:*:.* *** * :        * **:::

MG07677.1 	QKYTT-APVVNKAIGGRSARSYWNEGRFAEVANLVRAGDVVVIEFGHNDGGSP-NKNDNG
NCU09976.1	PPLLNGITVVNKAVPGRSARSYTNEGRFTELANLVTAGDIVVIEFGHNDGGSPRSASDNG
FG04848.1 	GKYLK-VNAVNKAIGGRSARSYTNEGRFQEIVNLVKPGDVVVIEFGHNDGGSP-NKNDNL
AN2528.1  	ADSVS-VEISNQAIGGRSARSYTREGRFQAIADVLQAGDYVVIEFGHNDGGSL--SNDNG
          	    .     *:*: ******* .****  :.::: .** ************    .** 

MG07677.1 	RSDCPGSGTEVCYSDKTG--EAVYTFVFYYIQAAKLMLAKGATVVLSSQTPNNLWEGGQY
NCU09976.1	RSDCPGTGSEVCVSGKTG--EKVYTFNHYVETAARAIIAKGASVIFSSQTPNNLWEGGSY
FG04848.1 	RSACPGAGTEVCTSDKTG--EKVYTYVFYISQAAKALVAKGAQVVLSTQTPNNQWETGTF
AN2528.1  	RTDCPGDGDETCETVYNGVAETVLTFPAYIENAALLFLEKGANVLISSQTPNNPWESGTF
          	*: *** * *.* :  .* :* * *:  *   **  :: *** *::*:***** ** * :

MG07677.1 	G-SGAP-RFVGYVATAQRALADS-RVTQVDHFTATARMYLKLGNVATNALYPRDHTHTSV
NCU09976.1	GGYGTPVRFVEYASLAAKNLAGTGKASFVDHYQAVANMYLKLGNAKTNSLYPSDHTHTSP
FG04848.1 	E-PGAP-RFVGYVSTAHRALASS-SVTWVDHFAAVTKMYQKLGNQKVNSLYPRDHTHTSP
AN2528.1  	S--YTPNRFVGYAELAAQRAGVD----YVDHGAYTASIFEALGADTVNSFYPNDHTHTNA
          	    :* *** *.  * :  .       ***   .: ::  **   .*::** *****. 

MG07677.1 	KGADLNAQSFAQAVAQGLNGTTSLKNLIISNYPRVY
NCU09976.1	EGADLVAKAFAQAVKLAWNGTTPLKGYLKAGNPTVF
FG04848.1 	EAADLIAKAFVQAINEEMNGRTSLKSLIKTPVSNVY
AN2528.1  	EGSSVVADAFLKAVVCSGVALNDVLTRTDFDGECL-
          	:.:.: *.:* :*:     . . :          :
```
